# Supplementary material for: Manual wheelchair biomechanics while overcoming various environmental barriers: A systematic review
Source: PLoS One. 2022 Jun 23;17(6):e0269657. doi: 10.1371/journal.pone.0269657 (PMC9223621; doi:10.1371/journal.pone.0269657)
Supplement: S1 Appendix — (DOCX) [file pone.0269657.s001.docx]

**Appendix: Biomechanical parameters definition**

**Spatio-temporal parameters**

***Stroke:*** propulsion cycle.

***Push phase:*** phase of a propulsion cycle during which the hand pushes on the handrim to propel the manual wheelchair.

***Recovery phase:*** phase of a propulsion cycle during which the hand is not in contact with the handrim.

***Contact angle:*** angle distance travelled by the hand on the handrim during the push phase.

***Push time:*** duration of a push phase.

***Recovery time:*** duration of a recovery phase.

***Cycle time:*** duration of a propulsion cycle.

***Cycle frequency:*** inverse of average cycle time.

***Speed:*** average speed of the manual wheelchair or average simulated speed of the manual wheelchair (on a roller ergometer or treadmill).

**Kinematics**

***Joint angle:*** angle between two skeletal segments.

**Kinetics**

***Rate of rise:*** represents the initial impact load on the pushrim. It has multiple definitions:

- Maximum value of the derivative of the resultant force with respect to time during the first third of the stroke (as defined in Koontz et al., 2005): $RoR=\max_{0\leq t\leq\frac{T}{3}} \frac{dF_{r}}{dt}$*, where RoR is the Rate of Rise, t time, T the duration of the stroke, and F_r_ the resultant force applied to the handrim.*
- Peak of the resultant force divided by the time to reach it since the beginning of the stroke (present in the literature, but not in our reviewed studies): $RoR=\frac{\hat{F_{r}}}{T_{peak}}$*, where RoR is the Rate of Rise,* $\hat{F_{r}}$ *the peak of the resultant force applied at the handrim, and T_peak_ the time to reach* $\hat{F_{r}}$*.*

***Handrim forces:*** forces applied to the handrim by the hand.

***Fraction of effective force:*** or mechanical effective force, represents the ratio between the forces useful to turn the rear wheel and the total force applied to the handrim. It has multiple definitions:

- Division of the square tangential force by the square resultant force (used in most of the reviewed studies): $FEF=\frac{{F_{t}}^{2}}{{F_{r}}^{2}}$*, where FEF is the Fraction of Effective Force, F_t_ the tangential force applied to the handrim, and F_r_ the resultant force applied to the handrim.*
- Propulsion moment squared divided by the handrim radius squared, all divided by the resultant force on the handrim squared: $FEF=\frac{\frac{M^{2}}{{r_{h}}^{2}}}{{F_{r}}^{2}}$*, where FEF is the Fraction of Effective Force, M the propulsion moment, r_h_ the handrim radius, and F_r_ the resultant force applied to the handrim.*

***Net joint moment:*** minimum moment required at a joint to obtain the observed kinematics.

***Mechanical power:*** product of handrim tangential forces and the manual wheelchair’s speed

***Mechanical work:*** time-integer of mechanical power
