# Supplementary material for: Manual wheelchair biomechanics while overcoming various environmental barriers: A systematic review
Source: PLoS One. 2022 Jun 23;17(6):e0269657. doi: 10.1371/journal.pone.0269657 (PMC9223621; doi:10.1371/journal.pone.0269657)
Supplement: Attachment — Submitted filename: Response to Reviewers.docx [file pone.0269657.s004.docx]

Responses to the reviewers’ comments

Dear reviewers,

Thank you for your encouraging comments while reviewing the manuscript and for allowing us to improve its content.

Please find below how we have addressed each of the reviewers’ comment. The reviewers’ comments are reported in black, whereas the answers are reported in blue.

A version of the revised manuscript has also been uploaded. Changes are highlighted in yellow in the revised version of the manuscript.

Kindest regards,

Théo Rouvier, on behalf of all the authors of the manuscript entitled "Manual wheelchair biomechanics while overcoming various environmental barriers: a systematic review”

***Reviewer 1:***

***General comments:***

This review gives a very good and extensive overview on the existing literature of manual wheelchair propulsion while overcoming different barriers in daily life of wheelchair users. The results are presented in detail in the tables which is very convenient for the interested reader to extract the information of interest. However, the presentation of the results in the text is sometimes lengthy and a repetition of what is presented in the table. This makes the review difficult to read. In my view, the review would benefit from shortening the text in the results section and referring to the tables instead. Detailed comments to the manuscript are written below.

Thank you for your appreciation and for your detailed review of our work. You will find hereafter responses to your detailed comments. Following your advice, the text in the “results” section was shortened and references to the tables were added.

**Introduction:**

Line 55: delete (MWU)

“(MWU)” was deleted from line 47 but the abbreviation was redefined at line 130 of the revised manuscript since it is used in the “results” and “discussion” sections.

Line 55-57: This sentence is not clear. What is the link between ICF and physical demands associated with barriers? Please rephrase.

We believe that ICF could be better implemented for MWC users if clinicians were able to adapt training programs in accordance with the difficulty of a barrier with regards to a user’s capabilities. The quantification of the physical demands associated with barriers could be used as a quantification of a barrier’s difficulty. The paragraph was rephrased. (l. 55-60)

Line 67-68: Sentence is not complete.

The sentence was rephrased.

Line 69: replace "in the same study" with "in each study".

This was corrected (line 67 of the revised manuscript).

Line 78-80: these two sentences are not well placed, they should be placed earlier in the introduction not just at the end where it's out of context.

The sentences have been moved up in the text at the beginning of the “Methods” section (lines 82-86 of the revised manuscript).

Line 80: to be specific, add "manual" to handrim propulsion

The adjective “manual” was added to be more specific, following the reviewer’s comment. (line 86 of the revised manuscript).

**Methods:**

Line 83: replace "consisted of" with "consisted in". There are other small language flaws in the manuscript, please let it check by a native English speaker.

The sentence was rephrased. The manuscript was checked by an English speaker and language flaws have been corrected throughout the manuscript.

Line 95-98: did you also try to include "inclination" as an addition to "slope"? This might have resulted in more studies.

Following the reviewer’s suggestion, we performed a new search on PubMed with the addition of the term “inclination”, which resulted in 21 new results compared to the initial PubMed search, among which some had already been retrieved with the initial Scopus search. Based on the screening on the title, most are related to seat or backrest inclinations, and not to the ground inclination (slope grade). Finally, only 1 reference appeared to be relevant, but it did not meet the inclusion criteria based on the screening on the abstract.

We agree with the reviewer that it remains possible that we missed some papers relative to very specific situations due to the absence of very specific keywords, but we believe that the global conclusions drawn on methodologies will not be challenged.

Line 118: S1 Appendix: The appendix does not include all parameters mentioned here, and not all parameters from the appendix are mentioned here. Please make it consistent.

Thank you for your comment, the parameters defined in the appendix and those mentioned in the “methods” section are now consistently the same.

**Results:**

Line 133: include a reference to table 1.

A reference to table 1 was added.

Table 2: van Drongelen 2005: information on kinematics is missing. I did not check the whole table on missing information, but just spotted this one. So please check again whether everything is included here.

Thank you for noticing this mistake. Information on kinematics recording in van Drongelen 2005 was added to Table 2. All the tables were double checked following your comment.

Table 3:	- what does * mean?

* Represents data that was extrapolated by the authors using the data given by the study. The signification of “*” was added to the tables, in the caption.

- what does sEMG mean?

sEMG means Surface EMG, this was detailed in the tables’ captions.

- Since it is a new table, explain the abbreviations below. Same accounts for the following tables.

All abbreviations are now defined in the caption of each table.

Table 5: why is in this table height and weight of the participants included, but not in the others? Keep it consistant over the tables.

It is true that data reported in tables was not consistent. We initially chose to report height and weight of subjects for the studies focusing on ground types because some of these studies normalized kinetic data on subject height and weight. But it is indeed confusing to report such data only for those studies and we don’t consider it crucial to the comprehension of our systematic review. For these reasons we normalized the tables and removed data on subject height and weight.

Line 156 and further: As already indicated in the general comments, all the information you give in here is already displayed in the table. The first part of the methods, where you summarize the range of participant number, the studied population and the range of slopes measured, is ok (although also not really needed since it is already indicated in the table). The second part on how the measurements were performed is obsolete, since you list again what is mentioned in the table (i.e. 19 articles measured kinematics, 15 article measured kinetics,..). Please shorten this part a lot or even delete it and refer to the table. The same accounts for cross-slope, curb and ground types.

Thank you for your comment. Following advice from both reviewers, the presentation of the experimental methods of the reviewed studies was shortened in the corpus of the manuscript. Full detail of the methods can be found in tables 2-5, and only key information was retained in the text.

Line 157: it should be referenced to table 2 instead of table 1.

This was corrected.

Line 187 and further: In general, I like the overview on the results in the appendix, it gives a good overview. If possible it would be good to take the tables out of the appendix and place it in the manuscript Then you could also shorten the text of the results part.

We agree with the reviewer’s that the tables provide a nice overview of the results, and, initially, it was our aim to place them in the manuscript.

However, given the lack of consensus in the literature on the outcome parameters of interest, it is difficult to provide tables that would fit over a page. The tables have indeed a large number of columns and few rows, which compromise their insertion in the text. Therefore, we chose to summarize the results in the paper and to refer to appendix table S2.

Line 187: Please refer to appendix Table S2, also further on in the results section.

We added a reference to the S2 Table in the slope sections, as well as in other sections (l.180-181 of the revised manuscript).

Line 188: replace "speed decreases with the grade of the slope" with "speed decreases with increasing slope"

The sentence was rephrased following the reviewer’s suggestion (l.184).

Line 193: replace "increase with the grade" with "increase with increasing slope"

The sentence was rephrased following the reviewer’s suggestion (l. 188).

Line 215: replace "increase in internal rotation with the slope" with "increase in internal rotation during propulsion on a slope"

This was changed to “increase in internal rotation with increasing slope”.

Line 241: should be referred to table 3 instead of 2.

The reference to the table was corrected. Thank you for spotting this error.

Line 250: before you always called it instrumented wheel instead of handrim dynanometer. Please use one of the labels conistently throughout the manuscript

Thank you for noticing this inconstancy. We replaced three usages (all of them) of “handrim dynamometer” by “instrumented wheel”.

Line 270: "on speed, cycle frequency and duration of push and recovery phase" can be deleted. In general, write more concisely and omit the information that is not really necessary or is a repetition. This makes it easier and more convenient for the reader.

Thank you for your comment which allowed us to increase the readability of our manuscript.

Line 274-275: give reference to the study and Appendix

Reference to the study and to the appendix were added (appendix reference l. 262-263 and study reference l. 268).

Line 296: should be referred to table 4 instead of 3.

This was corrected (l.293).

Line 296: "real" MWC sounds odd, please delete.

This was deleted.

Line 313: add reference to appendix.

A reference to the appendix table was given (l. 306-307).

Line 344: should be referred to table 5 instead of 4.

This was corrected.

Line 365: this is another example of sentences that are not needed, a reference to the appendix is more meaningful.

These sentences were deleted (reference to the appendix l. 352-353).

**Discussion**

Line 408: "investigated in literature" instead of "from literature"

The sentence was rephrased following the reviewer’s suggestion (l. 392).

Line 409-414: This information belongs to the results, there you already listed how many studies investigated which barrier. It does not have to be mentioned again, especially not with numbers of studies. You might want to say in one sentence what has been studied most.

The sentence was shortened following the reviewer’s suggestion (l. 393-394).

Line 431: safety belts were not used in all of the studies conducted on treadmills

Thank you for your comment. The sentence was changed, and now states that safety belts were sometimes used in studies conducted in treadmills (l. 444-447).

Line 443: Number of studies reporting kinematics and kinetics is for the results section, not for the discussion.

The sentence was shortened following the reviewer’s suggestion.

Line 467: include reference to the study.

The reference to the study was added (l. 451).

**References**

Reference 1: what kind of a reference is this? if it is a website, please indicate the url.

The reference was corrected to display authors, organisation, and website url.

Reference 11: Title is written twice in the reference

This was corrected.

Figure 1: indicate in the figure why the records (n=1098) they are excluded

The 1098 records were excluded after either a screening on title or abstract. These records were excluded based on our inclusion/exclusion criteria. These criteria are detailed in section (2.2 – Article selection) of the manuscript. “The inclusion criteria were original study or systematic review, study written in English and experimental work. Exclusion criteria were articles about electric wheelchairs, powered-assisted wheelchairs, sports wheelchairs, other propulsion systems than manual handrim, hemiplegia-pattern propulsion”.

It was added to the PRISMA flow-chart that these records were excluded after title or abstract screening.

Reasons detailing why studies were excluded in the full-text screening were also added to the flow-chart.

S2 Table: Check the table, units are not always indicated. For example, in Slope: MWC speed, recovery phase duration, contact angle, sometime it's indicated after the numbers instead of in the the title line (i.e. body kinetics), or both in the title line and after numbers (Peak un-normalized EMG). Please make it consistent.

Thank you for this comment. It is true the reporting of units was inconsistent. All units are now consistently reported. Units for all values except those that are normalized are reported in the title line. Units for values that are normalized are always reported in each cell, because studies did not always use the same normalization criterion, even on the same biomechanical parameter, and therefore had different units for the same parameters.

**Appendix**

- Contact angle: you define it as "angle distance travelled by the non-dominant hand on the handrim during the push phase". Why is it limited to the non-dominant hand?- Rate of rise: your definition is not correct, not detailed. Is it the mean resultant force divided by the contact time of the whole cycle?

Besides that, in literature there are different definitions:

• taking the derivative of FR with respect to time and then determining the maximum value during the first third of the stroke

• (first) peak of the resultant force, divided by the time to reach the peak.

Therefore, write it more precisely in your definition.

- Fraction of effective force: There are also different definitions used, either FEF = (Mwheel·r−1)·Ftot−1 or Ftan2/Fres2

Thank you for your comment, the definition of these biomechanical parameters was detailed. We presented both definitions for the two latter parameters, and indicated which one was more present in the reviewed articles.

***Reviewer 2:***

A nicely written systematic review, which presents the work in a very detailed and extensive way. In more detail:

Thank you for the appreciation of our work and your thorough review of the manuscript.

Introduction: the introduction is written well, only the ending could use some finetuning.

The last sentences of the introduction were moved up in the introduction so as to provide a better ending of the introduction, following the reviewer’s comment.

Methods: Methods section is well presented and only lacks a clear inclusion/exclusion overview.

Inclusion and exclusion criteria were separated in the Methods section following the reviewer’s suggestion.

Results: The results were separated for the four areas (slopes, cross-slopes, curbs and ground type), as well as methods and results.

Regarding the methods on slopes, cross-slopes, curbs and ground type: It is unnecessary to present all results on methods this extensively. Most information can also be found in the presented tables, which already cover a lot of information. Removing parts in these sections might help with the readability of the whole paper.

On the Results on slopes, cross-slopes, curbs and ground type part: Adding subheadings for the subcategories: spatio-temporal, kinematics, kinetics and muscle activity are recommended.

Thank you for your comment. Following your recommendations, the presentation of the reviewed methods in the “Results” section was shortened to only contain information we deemed key for the reader.

Also, as suggested, subheadings were added in the “Results” section for the different biomechanical parameters (spatio-temporal, kinematics, kinetics, muscle activity).

Discussion: In the discussion section a lot of important information is discussed. It feels like a lot of separate interesting discussion points are raised but the structure is a bit lacking. The transitions from one paragraph to another, as well as the general structure should use some finetuning.

Thank you for your comment, we have worked on improving the flow of the “Discussion” section. Paragraphs were re-arranged and subheadings were added to place a greater emphasis on the different key points addressed in the section. We think that this new general structure of the discussion improves its readability compare to the previous draft.

Conclusion: In this section a lot of information is discussed, which can either be removed or combined with some of the discussion points. Try to get the conclusion short and to the point.

Thank you for your comment and suggestion, the conclusion indeed felt too long. Points addressed in the discussion were either shortened or moved up into the “Discussion” section. The conclusion is now only two paragraphs short.

Smaller general points: The consistency in the use of references, sometimes all references are listed extensively, but there are cases in which some the references are missing, examples are Lines 380-382 and Lines 426-442.

Thank you for noticing these missing references. References were added in both paragraphs as suggested (l. 365, 443-45 of the revised manuscript).

Maybe a figure with the 4 environmental barriers might be a good addition to have a clear overview and explanation of the 4 barriers (cross-slope might be unclear to some naïve readers).

Thank you for your suggestion. Thank you for your comment. Pictures of the recreated environmental barriers were moved from the supplementary materials (S1-3 Figs) to the corpus of the manuscript, and fused in one figure

**Abstract:**

Page 2, Lines 33-35: Make the conclusion at the end a bit stronger, something similar to: “It is recommended to standardize the procedure, studying a wide set of…”

Following the reviewer’s comment, the concluding sentence of the abstract was rephrased to “It is recommended to standardize the procedure when studying various physical environmental situations and to systematically report MWC configuration(s). Furthermore, a wider set of situations should be studied” (l. 33-36).

***Introduction:***

Page 3, Lines 45-46: The sentence seems confusing, maybe something similar to: “Despite the improvement of overall accessibility of public areas…”

The sentence was rephrased following the reviewer’s suggestion (l. 45-47).

Page 3, Lines 46: Recommended to start a new paragraph when ICF is introduced

A new paragraph dedicated to the ICF was started (l. 48-60).

Page 3, Lines 61-62: Last sentence of this paragraph feels a bit redundant

Indeed, the last sentence was removed following the reviewer’s comment.

Page 4, Line 75: Remove the word “therefore” in this sentence

The word “therefore” was removed from the sentence.

Page 4, Lines 78-80: This paragraph is unnecessary, the end of the previous paragraph is a more proper ending of the introduction

The sentences of the last paragraph were not deleted as some studies focusing on other means of propulsion were retrieved from the literature and are not discussed in the present review. However, following both reviewer’s comments, they were moved in the Methods section. The introduction now ends on: ” To fill this gap, the purpose of this study was to identify and synthesize data and experimental methods from the literature on the biomechanics of MWC propulsion for various and frequent environmental barriers that are daily encountered by MWC users.” (paragraph moved to l. 82-86).

***Methods:***

Page 4, Line 83: Change the sentence a bit: “The present study conducted a systematic review to identify…”

The sentence was changed following the reviewer’s comment (l. 82).

Page 5, Lines 105-106: Remove “Appended to this paper…” and add “(S1 table)” add the end of “…(PRISMA) Checklist [24]”

This was changed (l. 108).

Page 5, Lines 106-110: Separate into inclusion/exclusion criteria, inclusion: biomechanical data, experimental work, English languages, exclusion: electric and power assisted wheelchairs etc.

The criteria for article selection into the present systematic review were separated into inclusion and exclusion criteria following the reviewer’s comment (see l. 109-112 of the revised manuscript)

Page 5, Line 111: Remove the word “three” from the sentence

Following the reviewer’s suggestion, “Three” was removed from the sentence as it was already cited earlier in the manuscript that three authors participated in the article selection process.

Page 5, Lines 114-116: Very nice and detailed description of the parameters

Thank you for your comment

Page 6, Line 117: Change towards: “A more detailed definition of the biomechanical parameters can be found in S1 Appendix”

The sentence was changed (l. 119).

Page 6, Line 118-119: Sentence feels a bit redundant

The sentence was moved to the section 2.1 of the manuscript. Indeed, it was a bit out of context in section 2.2 “Article selection” but it seemed relevant to the authors to insist of the methodological aspects of the studies as well as on the obtained results (l. 95-96).

***Results:***

Page 6, Lines 129-132: Split the sentence into two

The sentence was split into two sentences and a reference to Table 1 was added following both reviewers’ comments (l. 129-133).

Page 6, Lines 134-137: Cut the sentences a bit, similar to: “Experimental design, acquisition methods and measurement tools differed between studies. The MWC was propelled overground… …ergometer. Kinematics were either recorded…”.

The sentence was cut into three (l. 134-138).

Page 6, Lines 137-138: What is the use of “on the contrary”?

“On the contrary” was removed from the sentence.

Page 6, Lines 140-142: Try to rephrase the sentence a bit, it is unclear what is part of the tables and what of the supplementary material. Maybe similar to: “Table 1 represents the synthesis of all studies, the remaining tables (2-5) summarize the study review for the four categories (slope, cross-slope, curb, ground type). … are appended as supporting information (S2 Table)”.

Following the reviewer’s suggestion, the sentence was rephrased to clarify the organization of the review: “An overview of the retrieved studies is provided in Table 1. A dedicated subsection to each investigated environmental barrier (slope, cross-slope, curb, ground type) synthesizes the experimental methods used in these studies (also reported in Tables (2-5) ) as well as the obtained biomechanical results with summarizing the experimental method. A compilation of the studies' numerical results is appended to this document as supplementary material (S2 Table).” (l. 139-143).

Page 18-19, Methods on slopes: See general comment

As answered before in general comments, the presentation of the experimental methods of the reviewed studies was shortened. Full detail of the methods can be found in table 2, and only key information was retained in the text.

Page 19, Line 188-189: Sentence can be removed (“Results on cycle frequency…) and combined with the next sentence.

This sentence was removed.

Page 19, Lines 191-194: Try to shorten the sentence: “…increase with the grade in SCI subjects, but unaffected with AB subjects.”

The sentence was shortened as suggested.

Page 19: Lines 201-203: What kind of differences were found?

Thank you for this comment, we indeed did not detail which differences were found. A sentence detailing these differences was added to the manuscript: ”Contact angle was higher on the same slope when experimenting on AB subjects, and seemed to remain constant with different grades of slope in AB subjects, whilst contact angle tended to decrease with increasing slope in MWU.” (l. 198-200).

Page 20: Line 212: Remove the part “in the study where they were reported”.

This part was removed.

Page 20: Lines 219-220: Remove the part: “…in all studies reporting these data”.

This part was removed.

Page 21: Line 233: Change to past sense

The verb “give” was changed to the past tense, thank you for spotting that error.

Page 21-22, Methods on cross-slopes: See general comments

See general comments for response: the review of the studies’ methods was shortened in the text. While tables still give full detail on the methods of the reviewed studies, the text now holds only key information.

Page 22: Lines 266-267: Is an interpretation sentence, better be part of the discussion

Thank you for your suggestion. The sentence was moved into the discussion section (4.1) (l. 395-397 of the revised manuscript).

Page 22, Lines 270-274: Try to shorten this sentence, parts at the beginning of the sentence can be removed.

The sentence was rephrased (l. 265-268).

Page 23-24, Methods on curbs: See general comments

See general comments for response: the review of the studies’ methods was shortened in the text. While tables still give full detail on the methods of the reviewed studies, the text now holds only vital information.

Page 23, Line 296: Remove the word “real”

This word was removed.

Page 25-26, Methods on ground types: See general comments

See general comments for response: the review of the studies’ methods was shortened in the text. While tables still give full detail on the methods of the reviewed studies, the text now holds only vital information.

Page 27, Lines 380-382: The references are missing

References were added (l. 365).

***Discussion:***

Page 27-28, Lines 401-406: This paragraph only repeats the aims, without actually giving any relevant information, combining some of this information with the next paragraph might be a solution.

Thank you for your comment. The paragraph was shortened to avoid repetition and allows for a better introduction of the review’s focus on experimental design issues (l. 386-388).

Page 28, Lines 417-425: This part can be shortened a bit, there is some repetition, especially with the last sentence.

This part of the paragraph was split into two, creating one more paragraph to present our point better. The penultimate sentence of the part was changed to get rid of repetition (l. 406-415).

Page 29, Lines 428-430: Future and past sense are both used in this sentence

The sentence was changed so the verbs are conjugated accordingly (l. 417-418).

Page 29-30, Lines 443-454: The paragraph starts with summarizing the number of articles investigating kinematics/kinetics. Despite main focus of this paragraph seems on the differences in acquisition methods. Try to get the most relevant information at the start/end of the paragraph.

Thank you for this comment. Along with rewiever 1’s suggestions, the first sentence of the paragraph was shortened to skip the summary of articles investigating kinematics/kinetics (l. 471-472).

Page 30, Lines 469-476: This feels like a separate paragraph, since the main focus of this paragraph was on the use of instrumented measurement wheels on both side of the MWC.

Indeed, not starting a new paragraph felt odd. This was corrected and a new paragraph was created discussing joint coordinate systems usage (new paragraph l. 497-507).

Page 31, Lines 486-490: It is unclear why this sentence is presented in between brackets, would make the argument stronger to present it as an example.

Thank you for your remark, the part in the brackets was moved out of brackets to be used as an example (l. 529-531).

Page 32, Lines 505-508: Try to combine these sentences into one strong sentence.

These two sentences were combined into one (l.466-468 of the revised manuscript).

Page 32, Lines 509-516: This paragraph does not add too much to the whole review and could, feels like lose paragraph at the end of the discussion, see also general comments

The paragraph was moved up in the discussion, where it was more relevant (section 4.3) and was slightly reformulated (l. 508-517).

**Conclusion:**

Page 32-34: A conclusion is preferably short and to the point. The conclusion now consists of around five paragraphs in which also a lot of recommendations are given. Some of these recommendations are already discussed previously and don’t necessarily strengthen the paper.

Thank you for your comment. Following your recommendations, the conclusion was shortened and is now straighter to the point.

Page 32, Lines 519-524: The first paragraph summarizes/repeats what has already been repeated multiple times

The first paragraph was deleted.

Page 33, Line 526: This is one of the main lines to be used for the conclusion.

Page 33, Lines 530-536: This all can be said in one sentence in the discussion part.

The paragraph was moved to the discussion section and split into different sentences adding weight to their respective new paragraphs.

Page 33, Lines 537-540: Interesting point, also combine with some of the paragraphs in the discussion part (Future recommendation)

The paragraph was moved in the discussion, in the section 4.1 discussing the environmental barriers studied in the retrieved literature (l. 406-409).

Page 33, Lines 541-543: Repeating the fact that cross-slopes and curbs should be studies, but now focused on asymmetrical propulsion.

This paragraph indeed felt like a repetition of what was already discussed previously, for this reason, it was deleted.

Page 33-34, Lines 544-551: This seems like one of the main parts of the conclusion of the paper

This paragraph is now the conclusion’s and our manuscript’s end (l. 545-552 of the revised manuscript).
