# Supplementary material for: Manual wheelchair biomechanics while overcoming various environmental barriers: A systematic review
Source: PLoS One. 2022 Jun 23;17(6):e0269657. doi: 10.1371/journal.pone.0269657 (PMC9223621; doi:10.1371/journal.pone.0269657)
Supplement: Attachment — Submitted filename: Response to Reviewers.docx [file pone.0269657.s005.docx]

Responses to the reviewers’ comments

Dear reviewers,

Thank you for your encouraging comments while reviewing the revised manuscript and for allowing us to keep improving its content.

Please find below how we have addressed each of the reviewers’ comment. The reviewers’ comments are reported in black, whereas the answers are reported in blue.

A version of the revised manuscript has also been uploaded. Changes are highlighted in yellow in the revised version of the manuscript.

Kindest regards,

Théo Rouvier, on behalf of all the authors of the manuscript entitled "Manual wheelchair biomechanics while overcoming various environmental barriers: a systematic review”

***Reviewer 1:***

The authors have addressed my comments well and the manuscript is now much clearer and easier to read.
I only have some small comments to this revised version:
Thank you for your endearing comments. We will try to take the latter comments into account as best as we can.

**Abstract:**
In my view the conclusion stated in the abstract does not reflect the conclusion of your whole paper. Please include all recommendations given in the conclusion section (i-v), or state it more general. Furthermore, do you suggest to standardize the measurements or the reporting of it?
Thank you for your comment. We added the five recommendations made in the conclusion to the abstract. These recommendations include suggestions to both standardize the measurements (ii) and the reporting of it (i, iii, iv, v).

**Methods:**
Line 107: You refer to the PRISMA checklist when describing article selection. The prisma Checklist does not specify how to select articles but specifies what should be reported in a systematic review. So better refer to figure 2 or rewrite the first sentence.
Indeed, we should not have referred to the prisma checklist. The sentence now refers to figure 2, which was moved up in the manuscript to appear after this paragraph.

Line 110: You mention systematic review as a inclusion criteria, but another inclusion criteria is experimental work. If experimental work is a inclusion criteria, systematic reviews (as mentioned in the line above) will not be included.
Thank you for your comment. This error was fixed by modifying the phrasing of the inclusion criteria, which now state “features experimental results” instead of “experimental work”

**Results:**
Figure 2: In the description of figure 2 you indicate classification, but this is not addressed in figure 2.
Thank you for your comment. This was from an earlier draft of the figure. Classification was removed from the figure’s title.

Line 286: Please indicate whether EMG was measured on both sides or just on up-/ or downhill side.
EMG was measured only on the left side. This precision was added in the presentation of the methods and results of muscle activity in cross-slopes.

Line 344: include "wheel" after instrumented
This was corrected.

**Discussion:**
Line 440: include "due" in front of to.
This was corrected.

**Conclusion:**
Line 548-553: here you state what you recommend for further studies. Is this what you miss in the current studies, or is this all you would like to have reported in studies? And what for example about the participant characteristics?
Thank you for this question. These recommendations are based on what we miss in most studies of the literature, but also what we would like see reported in future studies. For example, some studies -but not all- reported barrier’s lengths and other characteristics, and we deem it is important that this practice be generalized. On the other hand, no reviewed study reported measurement uncertainties or model reconstruction errors, and we think it would be a truly interesting metric for researchers trying to compare their results with the state of the art. Participant characteristics are also a very interesting metric, but we feel that the state of the art has reached closer to a consensus, hence why we did not include them in our recommendations.

***Reviewer 2:***

Thank you for addressing all comments and the changes made to the manuscript. The changes have improved the readability of the manuscript. Only some minor adjustments are recommended.
A small note: Sometimes a tab is used to begin a new paragraph, sometimes not, there is no real consistency throughout the whole paper.
Thank you for your encouraging comments. Tabulations are now systematically used at the start of the first paragraph of a section.

**Abstract:**
Page 2, Lines 33-36: It would be recommended to make 1 strong final sentence: “It is recommended to standardize the procedure when studying various physical environmental situations, systematically report MWC configuration(s) and study a wider set of situations.”
Thank you for your comment. Alongside reviewer 1’s comments, we decided to end the abstract by the five recommendations listed in the conclusion. The sentence “Furthermore a wider set of situations should be studied” that was used to close the abstract was moved up to let our recommendations close the abstract.

**Introduction:**
The introduction now indeed ends with a logical flow towards the purpose of the study, well done.
Thank you very much for your comment.

**Methods:**
Page 5, Lines 109-112: The exclusion criteria are now nicely stated, only the inclusion criteria are still a bit too general (English language, original study, experimental work). It would be recommended to also add something similar to: “Studies investigating slope, cross-slope, curb and ground type in manual wheelchair users.”
Thank you for your comment. We changed Inclusion criteria and replaced “experimental work” by “features experimental results on slopes, cross-slopes, curbs, and ground types during MWC locomotion”.

**Results:**
Removing parts on the ‘methods’ section for all barriers improved the readability of the results part of the manuscript. Adding subheading really helped for the structure as well, great work.
Thank you for your comment.

Page 19, Line 180-182: These two sentences can be removed or moved a bit up, it is already previously stated on Page 7, Line 144 that the numerical results can be found in S2 Table. Now the same sentence is stated for all barriers on Page 22, Lines 263-264, Page 24, Lines 307-308 and Page 26, Lines 353-354. Stating that information once around line 144 will probably be enough, similar to: ‘Supplementary material on the detailed results on all environmental barriers (slopes, curbs etc.) can be found in S2 Table.’.
Thank you for your comment. Duplicates were removed, and the reference to Table S2 is now only listed in the overview of general results.

Page 22, Line 265: The subheading ‘3.2.2.1. Spatio-temporal parameters’ is too big, should be similar to the ‘3.2.2.2. Joint Kinematics’.
This was corrected.

**Discussion**
Page 27, Lines 387-389: This introductory statement might not necessarily be needed, in the next paragraph it is also summarized that environmental barriers were investigated.
The introductory statement was deleted.

Page 28, Lines 411-422: One nice combined paragraph can be made out of these three separate paragraphs with the last one only being one sentence.
Thank you for your comment. The three paragraphs were merged into one

Page 31, Lines 483-497: This has become a rather long paragraph to only state one thing: ‘Instrumented wheels should be used on both sides’. Especially lines 490-495 are repetitions of the first sentences of the paragraph.
Thank you for your comment. The paragraph indeed felt long. We shortened the paragraph to restrain from repeating ourselves, and focused the latter half of the paragraph on the fact that the only study that investigated cross-slopes using two instrumented wheels, reported results on the dominant and non-dominant sides of the user without expressing which one was downhill and uphill, which is also an important factor in the biomechanics of cross-slope locomotion

Page 32, Lines 509-511: Which studies are you referring to?
We added the precision as to which studies we are referring to: all studies presented in this review.

Page 32, Line 511: ‘… might have resulted in significant uncertainties’.
This was corrected

**Conclusion:**
The conclusion is a lot more shortened and to the point, well done.
Thank you for your comment.

Page 33, Line 538: The first sentence is a bit unclear, seems like there is missing some information.
The first sentence was rephrased: “This review highlighted discrepancies in focus given to each environmental situation in the literature”.

Page 33, Line 545: ‘… parameters retrieved from the relevant literature.’
This was corrected
